# Decoding the PTM code of cGAS–STING in gastric cancer: from innate DNA sensing to precision combination therapy

**DOI:** 10.3389/fimmu.2026.1881282

**Published:** 2026-07-01

**Authors:** Qiang Li, Yucheng Peng, Duanrui Liu, Xiumin Ma, Yufei Wu

**Affiliations:** 1 Clinical Laboratory Center, Tumor Hospital of Xinjiang Medical University, Urumqi, Xinjiang, China; 2 State Key Laboratory of Pathogenesis, Prevention and Treatment of High Incidence Diseases in Central Asia, Clinical Laboratory Center, Tumor Hospital of Xinjiang Medical University, Urumqi, Xinjiang, China; 3 Department of Clinical Laboratory, Shandong Provincial Hospital Affiliated to Shandong First Medical University, Jinan, Shandong, China; 4 School of Biological Sciences, The University of Hong Kong, Hong Kong, Hong Kong SAR, China

**Keywords:** CGAS, combination therapy, gastric cancer, post-translational modifications, STING

## Abstract

The cGAS-STING pathway represents a crucial component of innate immune responses and has been increasingly recognized as a pivotal regulatory mechanism governing gastric cancer progression and therapeutic resistance. This comprehensive review synthesizes current knowledge regarding the mechanisms through which Post-Translational Modifications (PTMs) modulate the activity, stability, and subcellular localization of cGAS and STING proteins, encompassing phosphorylation, ubiquitination, acetylation, SUMOylation, and palmitoylation. These PTMs function as pivotal regulators that maintain the delicate balance between immune activation and suppression within the tumor microenvironment, directly influencing tumor immune evasion, proliferation, and metastatic potential. Furthermore, we critically examine the dual role of the cGAS-STING pathway in gastric cancer, highlighting its context-dependent anti-tumor and pro-tumor effects. We also investigate potential therapeutic strategies targeting post-translational modifications to restore or potentiate cGAS-STING signaling, which could substantially enhance the efficacy of chemotherapy, radiotherapy, targeted therapy, and immunotherapy. Our comprehensive analysis underscores the substantial prognostic and therapeutic promise of PTM-directed interventions for gastric cancer management, providing a valuable foundation for future mechanistic research and clinical translation.

## 1 Introduction

Gastric cancer (gastric cancer) ranks among the most prevalent malignant tumors worldwide. According to the 2022 GLOBOCAN database, approximately 969, 000 new gastric cancer cases were diagnosed globally (1). In the treatment of gastric cancer, surgery is the main treatment of gastric cancer. As gastric cancer is often discovered at an advanced stage, surgical options are typically no longer feasible. Consequently, chemotherapy, radiotherapy, targeted therapy and immunotherapy have become the main treatments strategies (2, 3). However, these treatments are linked to considerable side effects and limitations. Therefore, there is an urgent need to discover new therapeutic targets to improve treatment effectiveness and extend patient survival.

Recent studies highlight the crucial role of the cGAS-STING pathway in the progression of gastric cancer, with its abnormal activation closely linked to tumor development, immune evasion, and resistance to therapy (4–6). The cGAS-STING signaling pathway plays a dual regulatory role, as it enhances immune defense against infections but may also lead to immune dysregulation (7). As a cytosolic DNA sensor, cGAS activates the production of interferon-beta (IFN-β) through a STING-dependent mechanism (8). This pathway affects various cellular processes, including autophagy, protein synthesis, energy metabolism, cell aggregation, DNA repair, cellular aging, and programmed cell death. It can also be regulated by multiple mechanisms, such as post-translational modifications of proteins. A deeper understanding of the regulatory mechanisms of this pathway could offer valuable theoretical insights and facilitate the development of clinical applications to improve cancer immunotherapy strategies (9, 10).

Post-translational modification (PTM) is the main mechanism for regulating the function of proteins. Many proteins, such as cGAS and STING, are precisely regulated through various covalent modifications like phosphorylation (11). These modifications are highly specific and can recruit different structural domains, including SH2 domains and other modules, ultimately coordinating signal transduction to influence biological activities. The intricate three-dimensional structure of cGAS-STING provides an ideal platform for multiple covalent modifications, which enhances our understanding of its regulatory roles in immune signaling (12). In gastric cancer, PTM related to the cGAS-STING pathway is deeply involved in the development and treatment response (13). PTM influences disease progression by reshaping the tumor microenvironment (14). In addition, PTM also can modify the activity of drug target proteins or affect drug sensitivity by regulating proteins in drug resistance-related pathways to promote chemotherapy and targeted therapy (15). These studies reveal that the protein modifications of cGAS-STING pathway play a crucial role in the progression of gastric cancer.

This article reviews the inducing factors, biological modification progress, therapeutic strategies, and possible treatment prospects of targeting the cGAS-STING pathway in gastric cancer. It aims to provide potential targets for the targeted therapy of gastric cancer and to lay a theoretical foundation for the early diagnosis, clinical prevention and treatment of gastric cancer.

## 2 The composition, regulation, and mechanism of the cGAS-STING pathway

**Figure 1:**
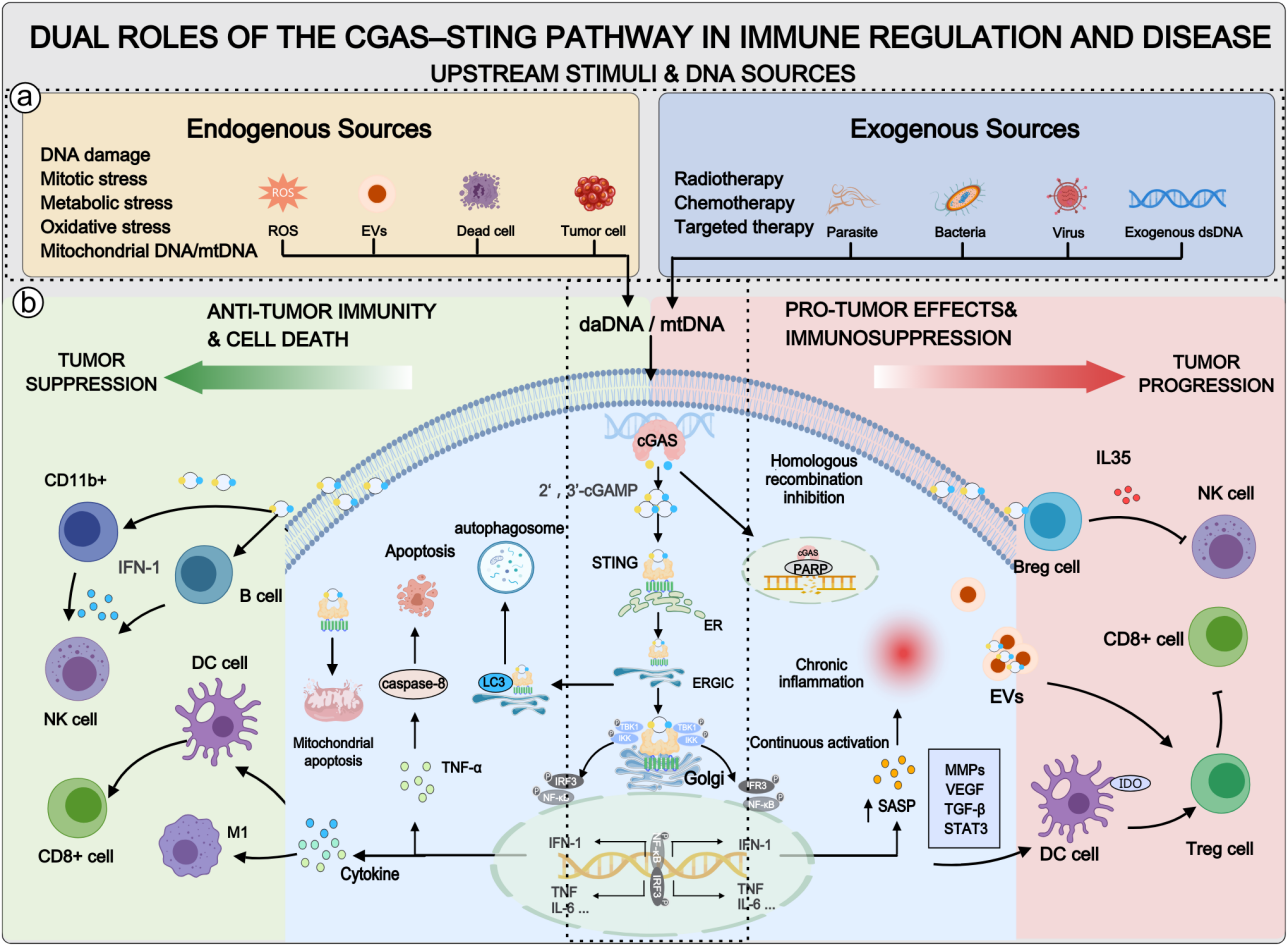
Dual roles of the cGAS–STING pathway in immune regulation and disease. **(a)** This schematic illustrates the cGAS–STING pathway’s dual role in disease. DNA damage, oxidative stress, and other stimuli activate cGAS, triggering STING and subsequent type I interferon production. **(b)** In the tumor context, this enhances immune activation and tumor suppression. However, chronic activation can induce immunosuppressive effects, including Treg/Breg cell expansion and inflammatory signaling, promoting tumor progression.

To clarify the complex mechanism of the cGAS-STING pathway in the progression of gastric cancer, it is necessary to analyze its core components, activation process and signal transduction (**Figure 1a**).

As a critical link between cytoplasmic DNA sensing and innate immune responses, the function of cGAS depends on a series of highly coordinated intracellular regulation (16). cGAS is responsible for recognizing cytosolic double-stranded DNA (dsDNA), which is the initial step in initiating the entire signaling pathway. Subsequently, cGAS catalyzes the synthesis of the second messenger cyclic GMP-AMP (cGAMP). cGAMP then mediates the activation of STING protein, which initiates downstream signaling pathways. This cascade promotes the activation of a series of transcription factors and ultimately triggering an immune response (17, 18). By studying how the cGAS-STING pathway is regulated and modified, we can understand its role in gastric cancer. This understanding will help develop new therapeutic strategies.

**Figure 2:**
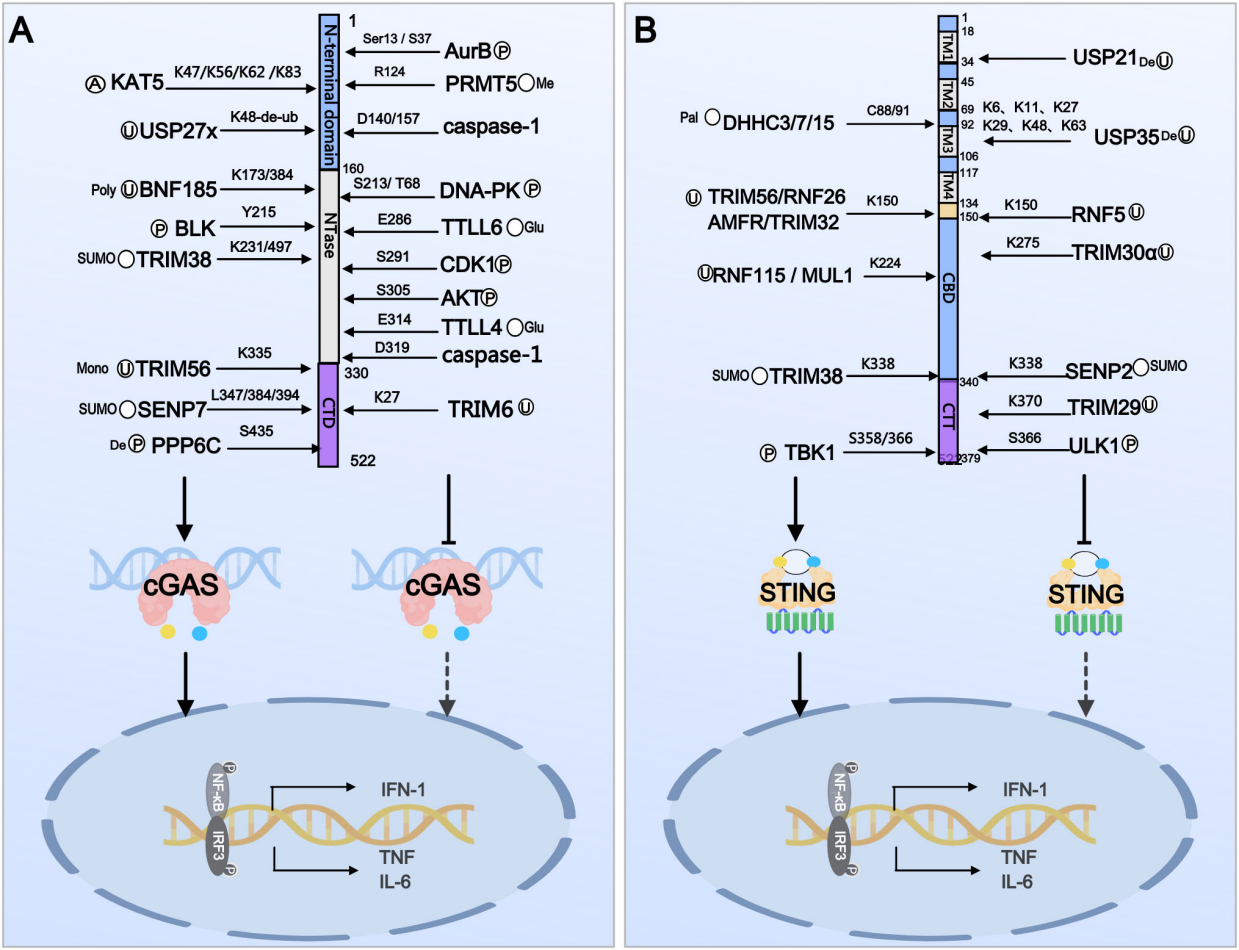
Regulatory post-translational modifications of cGAS **(A)** and STING **(B)** orchestrate the cGAS-STING inflammatory signaling cascade. **(A)** Domain organization of cGAS with annotated amino acid residues undergoing phosphorylation, ubiquitination, SUMOylation, methylation and other modifications, together with the corresponding catalytic enzymes. Modifications of cGAS control downstream IRF3/NF-κB-driven transcription of IFN-1, TNF and IL-6. **(B)** Schematic of STING structural domains and its multi-site post-translational modifications mediated by different enzymes, which govern STING-dependent production of inflammatory cytokines. Solid arrows denote signal activation, dashed arrows denote signal suppression.

The activity and stability of the cGAS-STING signaling axis are precisely regulated by PTMs, which is crucial for balancing immune activation and preventing autoimmunity (**Figure 2**). The key modifications of cGAS include Akt and DNA-PK mediated inhibitory phosphorylation, as well as acting ubiquitination (RNF185) (19). In addition, its function is finely regulated by acetylation (KAT5), SUMOylation (TRIM38, SENP7), glutamylation (TTLL4/6), caspase cleavage, and palmitoylation (ZDHHC18, ZDHHC9) (20). The regulation of STING is equally diverse. TBK1 phosphorylation promotes signal transduction, while multiple ubiquitination modifications (TRIM56/RNF26/USP21) collectively regulate its activity and stability (21). SUMOylation (TRIM38, SENP2) and palmitoylation (DHHC3/7/15) also profoundly affect STING function (20, 22). The competitive modifications present at specific sites form integrated regulatory hubs that jointly control the initiation, intensity, and termination of pathways, providing important theoretical basis for targeted therapy.

In tumors, the cGAS-STING pathway becomes highly activated. This activation is caused by genomic instability, mitochondrial DNA release, and therapeutic stress. These events lead to the accumulation of DNA in the cell cytoplasm. The pathway plays a complex role in tumor regulation (23, 24) (**Figure 1b**).

On one hand, it mainly mediates anti-tumor immunity. It does this by inducing type I interferon and other pro-inflammatory cytokines. This action promotes dendritic cell maturation. It also enhances the cytotoxic effects of NK cells and T cells and increases immune cell infiltration (25). On the other hand, its sustained activation can have opposite effects. It promotes an immunosuppressive tumor microenvironment (16). This occurs through the recruitment of regulatory T cells, the induction of immune cell death, and the facilitation of epithelial-mesenchymal transition. The balanced activity of key molecules like NF-κB and IDO is crucial in this process (**Figure 1b**).

There are several promising directions for future research and application of the cGAS-STING pathway in gastric cancer. One can focus on the site-specific regulation of PTMs to develop targeted agents that precisely modulate the activity of the pathway, addressing the issue of traditional pan-target intervention. Additionally, based on the functions of the pathway in the tumor microenvironment, personalized treatment strategies related to pathway activity and immune typing can be established to achieve precise treatment for different subtypes of gastric cancer. At the same time, the synergistic mechanism of pathway activators with radiotherapy, chemotherapy, and immune checkpoint inhibitors can be deeply studied to construct a combined treatment system that integrates tumor killing, innate immune activation, and adaptive immune enhancement.

## 3 Regulation of cGAS-STING in gastric cancer

**Figure 3:**
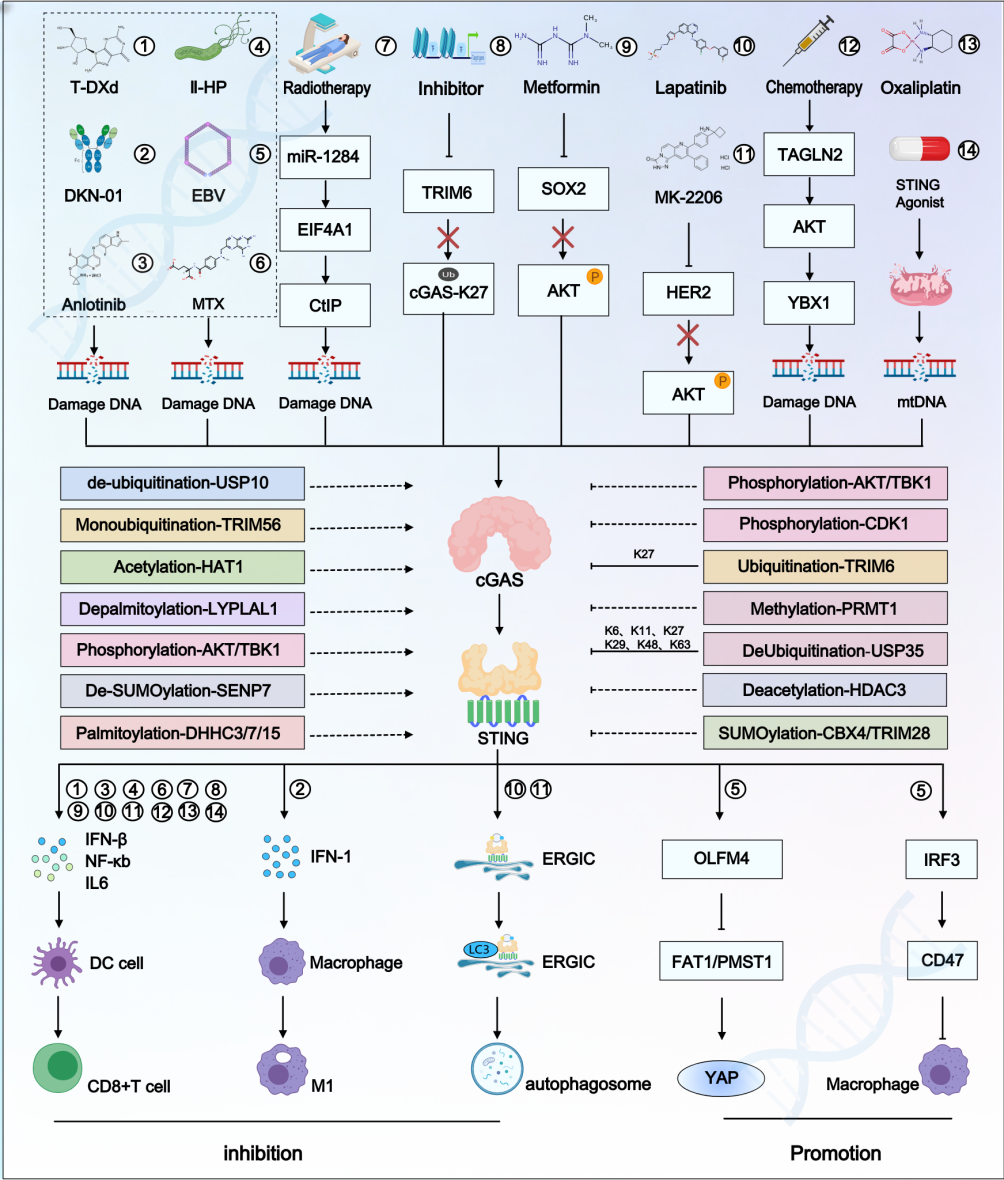
The regulation of cGAS-STING signaling pathway in gastric cancer. Multiple interventions (antibodies, small molecules, radiotherapy, STING agonist, etc.) modulate cGAS-STING via DNA damage, key molecules and PTMs. …:Not yet confirmed.

The main research on the cGAS-STING pathway in tumors is the cytoplasmic DNA-mediated immune response. In gastric cancer, it also involves specific mechanisms, including chronic Helicobacter pylori infection, specific regulatory mechanisms and protein modification changes. These factors disrupt the balance of the pathway between immune surveillance and immune escape, making it a key in the progression of gastric cancer (**Figure 3**).

### 3.1 Dysregulation of the cGAS–STING pathway is a participant in H. pylori-induced gastric carcinogenesis

Among the many environmental factors involved in the occurrence of gastric cancer, chronic infection of pathogens has always been regarded as a key driving factor. Helicobacter pylori (Hp) is the most characteristic pathogen among them and is a major risk factor for gastric malignancies (26, 27).

Hp can lead to the accumulation of DNA double - strand breaks (DSBs) and activate the cGAS-STING pathway (28). In gastric epithelial cells, organoids, and INS-GAS mouse mucosa, Hp upregulates the expression of ACVR1. ACVR1 activation inhibits POLD1, a key DNA repair enzyme, leading to DSB accumulation. Notably, the transcription factor IRF3, which promotes POLD1 activation, is suppressed by Hp (29). Additionally, Hp also evades innate immune sensing by downregulating IRF3 activation and inducing TRIM proteins to inhibit STING. The deficiency of STING enhances the acute inflammatory response to Hp within the gastric niche *in vivo* (30).

In gastric cancer progression, H. pylori infection not only provides a structural foundation for cGAS–STING pathway activation by inducing genomic instability and chronic inflammation but also may achieve immune evasion or reprogramming of this pathway through precise regulation of the host post-translational modification (PTM) network, thereby exerting dual pro- and antitumor effects within the tumor microenvironment (31). Research demonstrates that small ubiquitin-like modifier (SUMO) is conjugated onto lysine residues 335, 372, and 382 of cGAS, a modification that suppresses its DNA-binding capacity, oligomerization potential, and nucleotidyl-transferase activity in 293T and HeLa cells (32). H. pylori infection-induced oxidative stress (ROS) and the release of inflammatory cytokines can upregulate the expression of SUMOylation enzymes (SAE1, SUMO1, and SUMO2/3) (33), which may inhibit the activation of the cGAS–STING pathway, thereby potentially hindering the organism’s antitumor capacity to eliminate infected cells.

### 3.2 The influence of EBV on EBVa gastric cancer through the cGAS-STING pathway

EB virus has a close relationship with cGAS-STING pathway in gastric cancer, which affects the occurrence, development and immune microenvironment of gastric cancer through multiple mechanisms. Specifically, EBV infection leads to an increase in the expression of cGAS and the phosphorylation level of STING, which in turn regulates the expression of downstream target genes. On the one hand, EBV-activated cGAS–STING signaling stimulates CD47 expression (34). As an immune escape signaling molecule, CD47 can inhibit the phagocytosis of macrophages and help tumor cells evade immune surveillance. Blocking CD47 could further activate cGAS-STING signaling and promote anti-tumor immunity, which indicates that the PTMs of cGAS-STING plays a key regulatory role in CD47-mediated immune escape and immune activation. On the other hand, EBV infection induces OLFM4 expression via the cGAS–STING pathway (35). OLFM4 is the direct target gene of this pathway and can bind to the cadgroin domain of FAT1 through the secretion of large microbubbles (MVs). This interaction disrupts FAT1–MST1 binding, leading to YAP activation and promoting gastric cancer cell proliferation and tumor growth. These findings highlight the involvement of the cGAS–STING pathway in cellular signal transduction and tumor progression.

Furthermore, the cGAS–STING pathway is also implicated in remodeling the immune microenvironment in EBVagastric cancer. Expression levels of cGAS and STING are significantly elevated in EBVagastric cancer compared to EBV-negative gastric cancer and are correlated with increased infiltration of CD8^+^ T cells. The cGAS-STING pathway could induce type I interferon and the expression of T cell chemokines (such as CXCL9/10/11 and CCL5), which help to recruit CD8+ T cells and form an immunocompetent tumor microenvironment (36). Moreover, STING expression shows a positive correlation with PD-L1 levels in EBVagastric cancer. Both molecules are involved in immune checkpoint regulation and influence patient prognosis, suggesting that cGAS–STING signaling may modulate responses to immunotherapy via PD-L1 modulation (37).

In summary, EBV promotes gastric carcinogenesis through multiple mechanisms mediated by cGAS–STING activation, including facilitation of immune escape, regulation of proliferative signaling, and reorganization of the immune microenvironment. These findings highlight the potential of targeting this pathway for the treatment of EBVagastric cancer. Identifying the key post-translational modification sites of EBV-encoded proteins and the corresponding modifying enzymes in the future will enable us to fundamentally block the regulation of the cGAS-STING pathway by EBV. This will provide a new target for the development of EBV-specific therapeutic drugs and reduce treatment toxicity.

### 3.3 The regulatory mechanism and therapeutic association of the cGAS-STING pathway in HER2-positive gastric cancer

In HER2-positive gastric cancer, HER2 expression status is closely correlated with the activity of the cGAS–STING pathway, influencing innate immune function, tumor autophagy, and malignant biological behaviors including tumor migration and therapeutic response. Mechanistically, fundamental cellular experiments across multiple tumor types, including colorectal, breast, and epithelial cell models, have validated that the HER2 intracellular domain directly binds the C-terminal fragment of STING located on the endoplasmic reticulum and selectively recruits AKT1, rather than other AKT isoforms, to the STING signalosome complex (38). Functionally, the recruited AKT1 directly phosphorylates TBK1, ultimately attenuating STING phosphorylation, reducing transcription of IRF3 and NF-κB target genes, and limiting type I interferon and chemokine secretion. Existing gastric cancer cellular and clinical evidence has confirmed that elevated HER2 expression suppresses cGAS–STING signaling, accompanied by reduced STING protein abundance and elevated phosphorylated AKT levels in HER2-high gastric cancer lesions (39), findings consistent with the regulatory pattern deduced from pan-cancer foundational research. Accordingly, it is biologically plausible to hypothesize that the aforementioned HER2–AKT1–TBK1 axis is conserved in HER2-positive gastric cancer cells. In alignment with this regulatory logic, HER2 knockdown or pharmacological inhibition of HER2 in gastric cancer effectively restores cGAS–STING pathway activity, alleviates autophagy suppression, and restricts cancer cell migratory capacity (40).

Intratumoral HER2 heterogeneity results in uneven STING expression in clinical gastric cancer specimens, with lower STING levels and fewer CD8^+^ tumor-infiltrating lymphocytes observed in HER2-high lesions, while clinically elevated total STING predicts unfavorable gastric cancer prognosis (41). Correspondingly, HER2-targeted therapies, including T-DXd and small-molecule HER2 inhibitors, can reverse STING suppression. T-DXd induces cytosolic DNA accumulation via its TOP1-inhibiting payload to activate cGAS–STING signaling and enhance CD8^+^ T cell-mediated antitumor immunity, whereas small-molecule HER2 inhibitors disrupt the HER2–AKT1 axis to restore STING signaling and improve intratumoral immune infiltration (39, 42).

These findings indicate that further investigation focusing on the STING-related PTM landscape may help systematically elucidate HER2-mediated tumor immune escape in gastric cancer and provide novel theoretical foundation for optimizing combined antitumor regimens targeting the cGAS–STING pathway.

### 3.4 Mechanism of cGAS-STING protein modification in gastric cancer

Among various tumor types, the abnormal expression of PTM-regulated enzymes can affect the activity of cGAS and STING proteins by specific modifications to regulate tumor immune responses. These modifications include various forms such as phosphorylation, ubiquitination, and acetylation. These regulatory mechanisms are complex and have certain specificity. Currently, while specific ubiquitination and de-ubiquitination events have been directly confirmed in gastric cancer, investigations into other PTMs remain limited. Based on the results of studies on cGAS and STING modifications that have been reported in other types of diseases and in combination with the existing results in gastric cancer, it is reasonable to speculate that these modifications may have similar regulatory effects in gastric cancer(**Figure 3**).

#### 3.4.1 Confirmed PTMs in gastric cancer: ubiquitination and de-ubiquitination

The dynamic regulation of the cGAS–STING pathway in gastric cancer is intricately governed by specific post-translational modifications, with ubiquitination and deubiquitination emerging as pivotal regulatory events. Recent advances have systematically mapped the precise biochemical interactions, specific structural domains, and downstream metabolic consequences that determine the fate of these sensor proteins in gastric cancer.

Ubiquitin ligases function as critical negative regulators of innate immunity within the tumor microenvironment. A prominent example in gastric cancer is TRIM6, a RING-type E3 ubiquitin ligase that is markedly upregulated in microsatellite stable (MSS) and immunologically “cold” gastric cancers (43). Mechanistically, TRIM6 utilizes its PRY-SPRY domain to directly interact with the C-terminal catalytic region of cGAS while sparing the N-terminal DNA-binding domain. Upon binding, TRIM6 catalyzes the K27-linked polyubiquitination of cGAS—a process strictly dependent on its C43 catalytic site. This specific ubiquitin linkage serves as a potent degradation signal, triggering the proteasomal degradation of cGAS and subsequently dismantling the cGAS–STING–IFN-β signaling cascade. Consequently, TRIM6 depletion stabilizes cGAS, reactivates the innate immune response, promotes CD8^+^ T cell infiltration, and sensitizes refractory MSS gastric tumors to anti-PD-1/PD-L1 immune checkpoint blockade (ICB).

Conversely, gastric cancer cells exploit deubiquitinating enzymes to aberrantly stabilize pathway components, rewiring them to fuel malignant progression rather than immune clearance (44). USP35, a highly expressed deubiquitinase in gastric cancer, has been identified as a critical driver of disease progression (45). USP35 directly deubiquitinates and stabilizes STING; however, instead of eliciting canonical antitumor immunity, this stabilized STING constitutively activates the HIF-1α/FAK signaling pathway (46). This axis promotes aerobic glycolysis, providing essential metabolic energy to enhance the adhesion of gastric cancer cells to peritoneal mesothelial cells (PMCs). Furthermore, this pathological cascade extends beyond the intracellular compartment. Gastric cancer cell-derived exosomes carrying USP35 are secreted into the microenvironment and induce mesothelial-to-mesenchymal transition (MMT) in PMCs.

#### 3.4.2 Proposed PTM mechanisms based on extrapolation from other cancer types

Beyond the well-documented ubiquitin system, the structural conformation of cGAS and STING provides ideal platforms for an array of covalent modifications. Direct evidence of these PTMs occurring on cGAS–STING in gastric cancer models requires further biochemical validation. However, parallel evidence from other tumor types and the well-characterized enzymatic landscape of gastric cancer provide a compelling theoretical framework for future investigation.

##### 3.4.2.1 Phosphorylation

The hypothesis that cGAS and STING undergo regulatory phosphorylation in gastric cancer is supported by the established intracellular signaling dynamics of the tumor. As previously highlighted in the context of HER2-positive gastric cancer, the HER2/AKT1 axis may dampen antitumor immunity by recruiting AKT1, which directly phosphorylates TBK1 at serine residues, thereby impeding STING–TBK1 association and subsequent TBK1 K63-linked ubiquitination (39). AKT is consistently hyperactivated in gastric cancer (47) through mechanisms including HER2 amplification, PTEN loss, and H. pylori infection-induced oxidative stress (48, 49). Additionally, established virology and oncology models demonstrate that AKT directly phosphorylates cGAS–STING components, impairing DNA sensing (50, 51). Therefore, it is logical to extrapolate this mechanism to gastric cancer. It is plausible that the hyperactive AKT1 network not only interrupts downstream TBK1 activation but may also exert direct, multi-tiered inhibitory phosphorylation on cGAS and STING themselves.

In addition, the role of CDK1 in promoting tumor proliferation and inhibiting immune surveillance together promotes the progression and immune escape of gastric cancer (52, 53). CDK1, as a key driver of gastric cancer cell proliferation, can promote tumor progression. Moreover, CDK1 can phosphorylate cGAS at S291 site during mitosis in HT1080 cells, which may inhibit the ability of cGAS to sense DNA and block the activation of anti-tumor immunity. Research has found that in gastric cancer, the treatment with anlotinib can not only directly inhibit tumor proliferation by down-regulating the expression of CDK1, but also relieve the inhibition of cGAS by CDK1 (4). This restored its anti-tumor immune response and enhanced the effect of immunotherapy.

Phosphorylation has a dual regulatory effect on STING. In the classical pathway, phosphorylation at STING Ser358 is essential for TBK1 binding, and TBK1 then trans-phosphorylates Ser366, facilitating IRF3 recruitment and interferon production. However, ULK1-mediated phosphorylation at the same Ser366 site promotes STING degradation without activating IRF3 (54–56). Notably, metformin can reduce AKT phosphorylation by inhibiting the SOX2/AKT pathway, which in turn activates cGAS-STING signaling (13). Meanwhile, the inactivation of PTPs caused by Hp infection may indirectly enhance the phosphorylation activity of kinases such as AKT, further participating in the inhibition of the cGAS-STING pathway (57).

However, the current evidence regarding the direct phosphorylation of cGAS and STING in gastric cancer is still limited, and the specific regulatory details still require more research for verification.

##### 3.4.2.2 Methylation

Highly expressed PRMT1 in gastric cancer is closely correlated with poor patient prognosis; it suppresses the cGAS–STING pathway to reduce IFN-β secretion, thereby modulating macrophage polarization and facilitating tumor immune escape and progression (58). Knockdown or enzymatic inactivation of PRMT1 triggers DNA damage and cytoplasmic dsDNA accumulation in gastric cancer cells, activates the cGAS–STING cascade, and remodels the antitumor immune microenvironment. However, this study only identified an indirect regulatory mode of PRMT1 without verifying methylation modification on cGAS or STING proteins. Intriguingly, another study performed across multiple cell lines uncovered the direct molecular mechanism underlying PRMT1-mediated pathway regulation (59). It demonstrated that PRMT1 catalyzes asymmetric dimethylation at the conserved Arg133 residue of cGAS to impair cGAS dimerization and enzymatic activity, consequently abrogating downstream interferon signaling, whereas PRMT1 inhibition enhances antitumor immunity and synergizes with immune checkpoint blockade. Based on the conserved regulatory logic among epithelial malignancies and the phenotypic consistency between the two studies, together with the high evolutionary conservation of the cGAS Arg133 site across species, it is reasonably hypothesized that PRMT1 may repress cGAS–STING signaling in gastric cancer not merely indirectly via cytoplasmic dsDNA accumulation but also directly through Arg133 methylation of cGAS; importantly, such direct methylation-mediated repression may cooperate with indirect dsDNA-dependent regulation to collaboratively promote immune evasion in gastric cancer.

##### 3.4.2.3 SUMOylation

Evidence from non-gastric cancer models reveals that SUMO is conjugated onto specific lysine residues of cGAS (K335, K372, and K382) (23, 60). Crucially, in the context of gastric cancer, H. pylori infection-induced oxidative stress and the subsequent release of inflammatory cytokines are known to markedly upregulate core SUMOylation enzymes. By shifting the intracellular equilibrium toward this inhibitory SUMOylation, the H. pylori-infected tumor microenvironment may impair the activation of the cGAS–STING pathway. Consequently, future research should focus on targeting these H. pylori-induced SUMOylation networks and associated DNA repair imbalances, offering a rational strategy to reverse immune evasion and reinvigorate antitumor immunity in gastric cancer (33). Meanwhile, studies on the rs77447679 polymorphism of SUMO-associated genes in Chinese populations have demonstrated an association with gastric cancer risk, further suggesting that aberrant SUMO regulatory networks may participate in gastric cancer development by influencing cGAS–STING functions (61).

##### 3.4.2.4 Acetylation

The acetylation regulation of cGAS and STING is closely related to gastric cancer. The acetylation of cGAS is bidirectional. N-terminal acetylation mediated by KAT5 enhances its DNA binding ability and activate the immune response, while acetylation at specific C-terminal sites inhibits its activity (62, 63). HDAC3 can eliminate this inhibition through deacetylation (64). The expression of STING and its downstream signaling are regulated by HDAC3 etc. HDAC3 can reduce histone acetylation in its promoter region to down-regulate its expression and also affect pathway activation by regulating p65 acetylation (62). In gastric cancer, high expression of HDAC3 is a notable feature. It not only promotes tumor progression by activating pathways such as PI3K/Akt/mTOR, but also may abnormally catalytically deacetylation of cGAS inhibitory sites, leading to excessive activation of cGAS (65). These result in the deterioration of the tumor microenvironment through continuous inflammatory signals or the down-regulation of STING expression. This is consistent with the mechanism by which HDAC3 inhibits STING in endometrial cancer (66).

In addition, the abnormal expression of HAT1 in gastric cancer may inhibit the activity of cytoplasmic pathways by enhancing the nuclear translocation of cGAS, similar to the immune escape mechanism of HBV-related tumors (67). If the activated acetylation of cGAS by KAT5 is inhibited in gastric cancer, it may weaken the innate immune response. Additionally, its regulation of GPX4 may cross with the anti-oxidative stress function of cGAS-STING, and affect the survival of tumor cells together (68, 69). It is worth noting that the abnormal activation of SIRT2 in gastric cancer may inhibit the activation of cGAS by deacetylating G3BP1, which is consistent with its role as a negative regulator of the cGAS–STING pathway (70, 71).

In conclusion, the acetylation anomalies of cGAS and STING may be regulated through a multi-pathways network, such as HDAC3-STING axis, HAT1-cGAS nuclear translocation, SIRT2-G3BP1 inhibition. It is involved in the immune escape and malignant progression of gastric cancer, and its specific effect depends on the expression acetylase in the gastric cancer. These findings provide a theoretical foundation for mechanistic studies and immunotherapeutic strategies targeting HDAC3, HAT1, or SIRT2. In particular, intervention strategies for the acetylation state of cGAS-STING pathway are worthy of further exploration.

##### 3.4.2.5 Palmitoylation

The palmitoylation of cGAS and STING may play multiple roles in gastric cancer development by regulating innate immune signals and cellular metabolic homeostasis.

Palmitoylation is a post-translational modification that occurs at cysteine residues. It is catalyzed by catalyzed by zinc-finger DHHC-domain acyltransferases (ZDHHCs) (72). The palmitoylation of cGAS is catalyzed by ZDHHC18 at the C474 site. This modification reduces its binding affinity to DNA and suppresses its enzymatic activity and downstream innate immune responses (73). In gastric cancer, abnormal expression of ZDHHC18 may enhance cGAS palmitoylation to weaken the STING pathway and promote gastric cancer cell immune escape (74). This modification can be reversed by the LYPLAL1, and loss of LYPLAL1 function may further suppress cGAS activity (75).

STING is palmitoylated at Cys88 and Cys91 by DHHC3, DHHC7, and DHHC15. This modification is essential for recruiting TBK1 and IRF3 and activating innate immune signaling (76). Meanwhile, the palmitoylation of STING is involved in the interaction of mitochondrial VDAC2. If this process is abnormal, it may promote the survival of gastric cancer cells by regulating mitochondrial function (77). In gastric cancer, the decreased expression of ZDHHC2 is associated with lymph node metastasis and poor prognosis of gastric adenocarcinoma. Acyltransferases containing DHHC can promote the growth of gastric cancer by targeting Nrf2 signaling, and targeting cGAS de-palmitoylation can enhance the response to anti-tumor immunotherapy. It is suggested that the palmitoylation of cGAS and STING may be involved in the immune escape and progression of gastric cancer through the regulation of DHHC family enzymes. Its modified state may be a potential target for prognosis and treatment of gastric cancer (78, 79).

Current treatments for gastric cancer are difficult to precisely regulate the dynamic signals within the tumor, while research on post-translational modifications of proteins is driving treatment from “external inhibition” to “internal regulation”. The abnormal PTM in the cGAS-STING pathway within gastric cancer promotes immune escape and progression, and the abnormal expression of key modifying enzymes is the core driving factor. In the future, it is necessary to deepen the direct evidence of the mechanism of gastric cancer-specific PTM and explore combined intervention plans.

## 4 cGAS-STING pathway in gastric cancer treatment strategies and prospect

**Figure 4:**
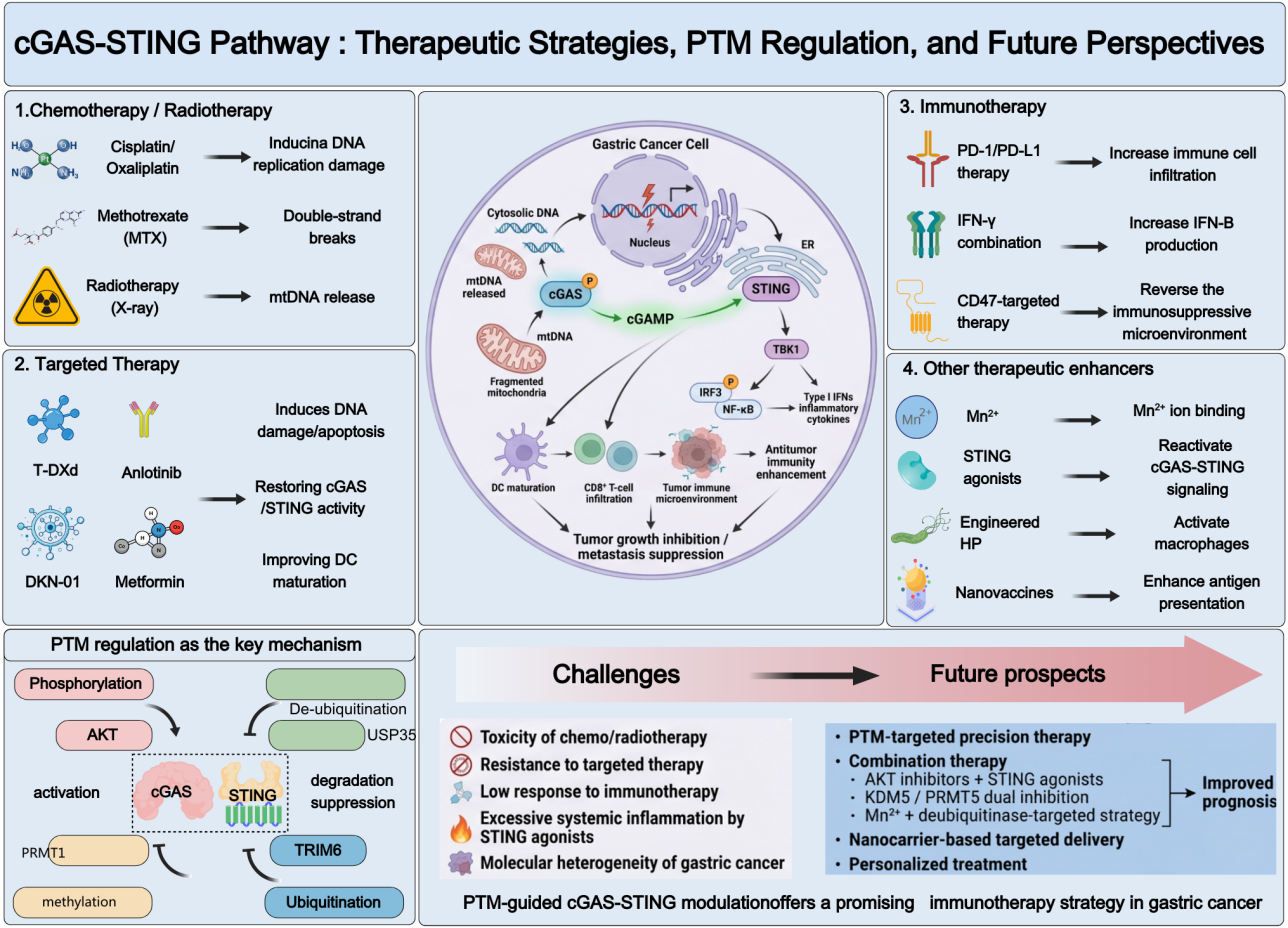
Treatment methods and possibilities in gastric cancer. This schematic highlights the therapeutic significance of cGAS–STING pathway modulation in gastric cancer. Conventional, targeted, and immunotherapeutic approaches can enhance cGAS–STING signaling and antitumor immunity. It also emphasizes the regulatory role of post-translational modifications, such as phosphorylation and ubiquitination, as potential targets for precision therapy. Despite challenges including therapeutic resistance and limited immune responsiveness, cGAS–STING-based strategies remain promising for personalized treatment in gastric cancer.

In the field of tumor immunotherapy, the cGAS-STING pathway, as a key signaling axis of innate immune response, has become an important target for enhancing anti-tumor immunity. Therefore, regulation of this pathway represents a promising and effective emerging strategy for cancer treatment. Currently, there are no clinical trials specifically targeting post-translational modifications of the cGAS-STING pathway in gastric cancer. We have listed ongoing clinical trials of STING agonists that target the cGAS-STING pathway, mostly conducted in other solid tumors. Although these trials are not specifically designed to address PTMs, they provide a reference framework for the future incorporation of PTM biomarkers into stratification strategies(**Supplementary Table 1**). We will also analyze the latest progress in gastric research and the potential application prospects in the future to provide theoretical support for the multimodal treatment of gastric cancer (**Figure 4**).

### 4.1 Current treatment approaches targeting cGAS-STING in gastric cancer

#### 4.1.1 Chemotherapy and radiotherapy

In chemotherapy, cisplatin and other drugs can destroy DNA replication or induce double-strand breaks in gastric cancer cells to release damaged DNA into the cytoplasm and activate the cGAS-STING pathway. For example, oxaliplatin induces immune cell death by opening the mitochondrial permeability transition pore (mPTP) to release mitochondrial DNA and activate the cGAS-STING-TBK1-IRF5 pathway (80). Low-dose methotrexate (MTX) specifically causes DNA damage in gastric cancer cells, directly activating cGAS–STING and promoting cGAMP production. It also sustains pathway activity by inhibiting ENPP1 to prevent cGAMP hydrolysis and adenosine generation (5). In addition, radiotherapy induces DNA damage in gastric cancer cells through X-rays, and the released DNA can also activate this pathway. This enhances immune recognition of tumor cells and synergizes with radiation to inhibit tumor growth. Studies have shown that combining MTX with radiotherapy enhances treatment efficacy and reduces metastasis (5). Although chemotherapy and radiotherapy can activate cGAS-STING by inducing DNA damage, its toxicity in gastric cancer is great and the survival benefit is limited. A study shows that the median overall survival period of patients undergoing radiotherapy and chemotherapy is 36 months, and the 3-year overall survival rate is 50% (81). Its effect is not ideal may be attributed to PTM-mediated drug resistance and immunosuppression. For example, high activity of AKT phosphorylation (incidence of gastric cancer >28.9%) may inhibit the activation of cGAS, leading to insufficient infiltration of CD8^+^T cells (82). Therefore, PTM-mediated drug resistance and immunosuppression may be the key factors that limit the ideal efficacy of chemotherapy and radiotherapy in gastric cancer by activating the cGAS-STING pathway.

#### 4.1.2 Targeted drug therapy

A variety of molecular targeted drugs play an anti-gastric cancer role by regulating the cGAS-STING pathway. T-DXd activates this pathway by inducing DNA damage and apoptosis. In HER2-positive gastric cancer, T-DXD enhances DCs maturation and CD8^+^T cell-mediated cytotoxicity by inducing IFN-I responses (39). In addition, DKN-01 targets DKK1 and inhibits tumor growth by activating this pathway to block macrophage polarization to the M2 phenotype (83). The combination of anlotinib and anti-PD-L1 antibody can enhance pathway activation and T-cell infiltration (4). It is worth noting that Metformin can mediate the inhibition of AKT phosphorylation by the transcription factor SOX2 to inhibit the phosphorylation of cGAS and STING (13). Capivasertib is a highly effective and selective targeting drug that inhibits three subtypes of AKT1/2/3. It was approved by the US FDA in November 2023 for the treatment of adults with HER2-negative advanced breast cancer, whose tumors carry mutations in PIK3CA, AKT1 or PTEN genes. This drug may also inhibit the phosphorylation of cGAS or STING to suppress tumor progression, particularly in HER2-positive gastric cancer (84). These findings indicate that molecular targeted drugs can affect the development of gastric cancer by regulating the post-translational modification of cGAS-STING.

#### 4.1.3 Immunotherapy

In immunotherapy, the efficacy of immune checkpoint inhibitors (ICIs) such as PD-1/PD-L1 antibodies are often limited by the high heterogeneity of gastric cancer. However, activation of the cGAS–STING pathway can enhance immune cell infiltration and remodel the tumor immune microenvironment through the induction of cytokines such as type I interferons (IFN-I). For instance, when IFN-γ is used in combination with STING agonists and PD-1 blockers, it can enhance the expression of cGAS and STING in cancer cells and promote the production of IFN-β and cell apoptosis (85). CD47-targeted immunotherapy represents another promising strategy, particularly in Epstein-Barr virus-associated gastric cancer. STING agonist ADU-S100 and diABZI combined with PD-1 antibody showed synergistic effect in colorectal cancer, which may enhance the reversal effect of ICIs on immunosuppressive microenvironment in gastric cancer (86). In addition, engineered low pathogenic Helicobacter pylori (Hp-Ce6) can serve as an oral immunomodulator that accumulates in tumors and activates the cGAS–STING pathway, thereby remodeling the macrophage phenotype (87). Nanovaccines based on peptide hydrogels or HPPS (high-density lipoprotein–peptide simulators) further enhance antigen presentation and antitumor immunity through coordinated activation of the cGAS–STING pathway and TLR9 signaling (88).

#### 4.1.4 Other therapies

In addition, Mn^2+^ can activate this pathway by enhancing the DNA perception ability of cGAS and the binding affinity of cGAMP to STING. In gastric cancer, Mn^2+^ can be used as an adjuvant in combination with vaccines or radiotherapy and chemotherapy. This helps promote the maturation of DCs and the infiltration of CD8^+^T cells to enhance the immune response (89). Demethylating drug can reactivate the cGAS-STING pathway in gastric cancer cells (90, 91). When it was used in combination with cGAMP, it can significantly inhibit tumor growth and prolong survival. Furthermore, during disulfidptosis, the accumulation of cysteine within cells leads to the abnormal formation of a large number of disulfide bonds. STING contain crucial cysteine residues and provide a structural basis for this modification (92, 93). Therefore, this modification may become a potential mechanism for the regulation of gastric cancer.

### 4.2 Post-translational modifications of the cGAS-STING pathway as precision therapeutic targets in gastric cancer

#### 4.2.1 Activating the cGAS-STING pathway inhibits the development of gastric cancer cells

On one hand, cGAS inhibitors suppress aberrant signaling by competing for enzymatic active sites or obstructing dsDNA binding (94). Representative inhibitors, such as PF-06928215, RU.521, perylaldehyde, and quinacillin, show promise for treating autoimmune and neurodegenerative disorders (95, 96). STING inhibitors primarily exert their inhibitory effects through covalent modification of cysteine residues within the transmembrane domain (97) or by competitively occupying ligand-binding sites (98, 99). These actions inhibit the palmitoylation or oligomerization of STING. Additional compounds, such as UNC93B1, act by promoting the degradation of the STING protein (100). The majority of currently identified STING inhibitors remain in the preclinical evaluation phase, and their role in inhibiting the persistent activation state of cGAS-STING in tumor treatment remains to be investigated.

On the other hand, STING agonists have demonstrated potential in the field of tumor treatment and have been explored in various tumor types (**Supplementary Table 2**). Notably, studies in gastric cancer have revealed that low expression levels of STING are associated with poorer patient prognosis (101). The knockout of STING in gastric cancer cells has been shown to promote more aggressive malignant behaviors, suggesting that activating the STING pathway could serve as a promising strategy to inhibit gastric cancer progression. Since STING agonists can change the immunosuppressive environment of tumors, they might also work in gastric cancer. For example, in gastric cancer, diABZI may exhibit antitumor activity via the phosphorylation of STING, similar to its observed effects in colorectal cancer (102). However, the antitumor efficacy of these interventions may be mediated through multiple parallel mechanisms; therefore, attributing the therapeutic effects solely to cGAS–STING activation would be incomplete.

#### 4.2.2 Precision PTM targeting: overcoming the limitations of generic agonism

The conventional approach to activating the cGAS-STING cascade relies on pan-STING agonists (e.g., diABZI, ADU-S100) or generic cGAS activators. While promising in specific settings (such as overcoming the immune desertification in Epstein-Barr virus-associated gastric cancer when combined with CD47 or PD-1 blockades (102)), direct systemic stimulation runs the severe risk of triggering excessive inflammatory responses, tissue damage, and narrow therapeutic windows. Moreover, these exogenous agonists blindly push the accelerator while ignoring the intrinsic “molecular brakes” (inhibitory PTMs) applied by the tumor cells. Therefore, precision interventions targeting specific PTM-modifying enzymes, particularly within the ubiquitination cascade, represent a paradigm shift.

Protein post-translational modifications, particularly the highly dynamic ubiquitination and deubiquitination cascades, serve as master regulators governing the cGAS–STING pathway. Recent evidence demonstrates that the aberrant PTM landscape in gastric cancer not only dictates the pathway’s stability but also determines its antitumor or pro-tumor fate. In this context, conventional pan-STING agonists may be limited, as they do not address the intrinsic molecular brakes applied by tumor cells and pose risks of systemic toxicity (103). Therefore, utilizing small interfering RNA (siRNA) or short hairpin RNA (shRNA) therapeutics to precisely silence specific E3 ubiquitin ligases or deubiquitinases emerges as a rational and promising precision medicine strategy.

The rationale for targeting PTM modulators via RNA interference can be stratified into two critical dimensions based on the contextual dual role of the cGAS–STING pathway: reversing immune evasion and halting metabolic-driven metastasis.

From a therapeutic perspective, establishing small nucleic acid drugs targeting TRIM6 holds transformative potential. By systematically knocking down TRIM6 expression, the K27-linked ubiquitination of cGAS is abrogated, restoring cGAS protein stability and reactivating endogenous antitumor immunity. Preclinical data demonstrate that TRIM6 ablation restores CD8^+^ T lymphocyte infiltration. Crucially, because the efficacy of anti-PD-L1 therapy intrinsically depends on downstream cGAS signaling, TRIM6-targeted siRNA could serve as a sensitizing agent. Administering TRIM6-siRNA in combination with ICB may effectively convert the phenotype of MSS gastric tumors from immunologically “cold” to “hot, ” offering a pharmacological breakthrough for ICB-refractory patients.

Conversely, the cGAS–STING pathway represents a biological paradox: under specific stress conditions, its overactivation may function as an oncogenic driver (104). This is particularly evident in gastric cancer peritoneal dissemination, where the deubiquitinase USP35 dictates a pro-tumorigenic PTM profile. This mechanistic insight suggests a distinct therapeutic strategy for advanced, high-metastatic-risk gastric cancer. Utilizing shRNA or siRNA therapies to silence USP35 may deprive STING of its deubiquitination protection, leading to its rapid ubiquitination and degradation. This targeted intervention may precisely sever the pathological signaling loop: it disrupts HIF-1α/FAK-mediated metabolic reprogramming and reverses the aberrant glycolytic phenotype. Therefore, USP35-targeted interference drugs represent a promising prophylactic or therapeutic intervention for patients susceptible to peritoneal metastasis.

### 4.3 The cGAS-STING-modified therapeutic approaches may improve the prognosis of patients with gastric cancer

The treatment of gastric cancer still has limitations (105). Chemotherapy and radiotherapy have side effects. Targeted drugs have drug resistance and narrow applicability. Immunotherapy has a low response rate and immune escape. STING agonists may trigger an excessive systemic inflammatory response, especially causing damage to normal tissues at high doses (106). Strategies aimed at modulating post-translational modifications (PTMs) of the cGAS–STING pathway hold broad promise. The key is to accurately control the PTMs in this pathway. This balances its ability to activate anti-tumor immunity while preventing immune suppression from overactivation, leading to a safe and effective treatment.

The contrasting functions of TRIM6 and USP35 illustrate the need for precise PTM modulation. Small interference drugs possess the mechanistic precision required to target these specific modifying enzymes. By deploying individualized RNA interference therapeutics that target specific nodes—for example, silencing E3 ligases such as TRIM6 to prevent immune evasion or inhibiting deubiquitinases such as USP35 to arrest metastasis—we can modulate the intracellular PTM rheostat and balance the cGAS–STING signaling threshold. This strategy may bypass the side effects of systemic agonists and synergize with existing multimodal therapies such as ICB. Ultimately, interventions guided by the PTM profile of individual gastric tumors may provide a theoretical foundation supporting the achievement of precision immunotherapy and substantial improvement of patient prognosis.

Combination therapies will further expand their potential applications. Current research shows that the synergistic effect of the STING agonist ADU-S100 with PD-1/PD-L1 inhibitors can reshape the tumor microenvironment (86). Such modification-based combination strategies may offer more precise treatment. Additionally, HDAC3 inhibitors can restore STING expression by relieving acetylation suppression at its promoter (66), and when used with cGAMP, they can enhance pathway activation while avoiding systemic inflammation that might be triggered by single agonists. Manganese ions (Mn^2+^), which naturally activate the cGAS-STING pathway, can improve cGAS-DNA binding and enhance the interaction between cGAMP and STING (107). When Mn^2+^ is combined with drugs targeting de-ubiquitinases USP14/USP27x (108), it stabilizes cGAS protein levels and further enhances immune responses.

However, multiple challenges must be addressed in the development of targeted therapies aimed at modifying the cGAS–STING pathway. dMMR/MSI-H and EBV-positive tumors tend to exhibit higher tumor-intrinsic cGAS–STING activity and greater immune infiltration, whereas MSS and HER2-positive settings may be more immunologically suppressed. PTM-targeted strategies are therefore unlikely to behave uniformly across gastric cancer subtypes. A modifier that restores cGAS stability in immunologically cold MSS gastric cancer may be beneficial, whereas indiscriminate pathway activation in an already inflamed setting could increase toxicity or generate non-productive inflammation. Another unresolved issue is that most candidate interventions act broadly rather than selectively. HDAC inhibitors, AKT inhibitors, PRMT inhibitors, and palmitoylation inhibitors regulate numerous substrates beyond cGAS or STING, making it difficult to attribute therapeutic responses solely to the cGAS–STING pathway. Furthermore, many studies rely on cell lines or xenografts, and direct validation of PTM sites in human gastric cancer tissues is rare. These limitations should be explicitly acknowledged because they constrain clinical translation. Finally, an important methodological gap remains: few gastric cancer studies integrate phosphoproteomics, ubiquitinomics, acetylomics, or SUMO-proteomics with functional immune readouts. Future work should prioritize residue-level PTM mapping in gastric cancer specimens, define subtype-specific PTM signatures, and test whether these signatures predict response to immune checkpoint blockade, chemotherapy, or radiotherapy. Such studies are necessary before PTM-directed modulation of cGAS–STING can be considered a mature therapeutic strategy in gastric cancer.

## 5 Conclusion

This review discusses how abnormal PTMs of cGAS and STING in gastric cancer leads to dysregulation of signaling pathways and regulates immune evasion, tumor progression, and therapeutic resistance. It emphasizes that PTMs, including phosphorylation, ubiquitination, acetylation, SUMOylation, and palmitoylation, are key regulatory factors for cGAS-STING activity. Precise regulation of their activation, signal transduction, and degradation can balance the immune response within the tumor microenvironment. We also outline that targeting the cGAS-STING pathway is expected to exert a synergistic effect with chemotherapy, radiotherapy, targeted therapy, and immunotherapy for gastric cancer. Therefore, exploring the regulation of cGAS-STING mediated by PTMs in gastric cancer and its immune escape mechanism can accumulate theoretical basis for subsequent basic research and innovative immunotherapy strategies.

## Supplementary material

Supplementary Table 1 Combination therapy targeting the cGAS-STING pathway.

Supplementary Table 2 Clinical drugs for cGAS-STING pathway.
