# Supplementary material for: Decoding the PTM code of cGAS–STING in gastric cancer: from innate DNA sensing to precision combination therapy
Source: Front Immunol. 2026 Jul 1;17:1881282. doi: 10.3389/fimmu.2026.1881282 (PMC13369448; doi:10.3389/fimmu.2026.1881282)
Supplement: Supplementary Table 1 — Combination therapy targeting the cGAS-STING pathway. [file Table1.docx]

**Decoding the PTM Code of cGAS–STING in Gastric Cancer: From Innate DNA Sensing to Precision** **Combination Therapy**

**Qiang Li^1, 2^,** **Yucheng Peng^1^, Duanrui Liu^3, 4^, Xiumin Ma^1, 2*^****and Yufei Wu^1, 2*^**

**Supplementary Table 1 Combination therapy targeting the cGAS-STING pathway**

| **Compound** | | **Indication** | **Combined therapy** | **Intervention/**  **Treatment** | **Phase** | **NCT** |
| --- | --- | --- | --- | --- | --- | --- |
| Ulevostinag | Head and Neck Squamous Cell Carcinoma | Ulevostinag + Pembrolizumab | IT；540 μg weekly on Day 1 for Cycles 1–2, then Day 1 of every 3‑week cycle for Cycles 3–35；up to ~2 years | Ⅱ | NCT04220866 |  |
| TAK-676 | Advanced/metastatic solid tumors | Dazostinag + Pembrolizumab +Chemotherapy | IV; Administer once on the first day of each cycle and the starting dose is 0.01–0.1 mg/kg. | Ⅰ/Ⅱ | NCT04420884 |  |
| SB11285 | Advanced/metastatic solid tumors | SB11285 + Atezolizumab | IV; Monotherapy and combination therapy groups received intravenous infusions once on days 1, 8, 15, and 22; 2 mg per dose. | Ⅰ | NCT04096638 |  |
| BI1387446 | Advanced/metastatic solid tumors | BI1387446 + Ezabenlimab | IT ; A 4-week period constitutes one cycle. Intratumoral injection of 0.01-1.0 mg/time on the first day of each cycle | Ⅰ | NCT04147234 |  |
| IMSA101 | Metastatic Kidney Cancer | IMSA101 + Nivolumab + PULSAR | SOC treatment: Nivolumab 480mg monthly PULSAR: 36 Gy in 3 fractions, Q4weeks IMSA101: three intra-tumoral injections of one of the progressive lesions at 1200 mcg (C1D1, C2D1, C3D1) | Ⅱ | NCT06601296 |  |
|  | Refractory Malignancies | IMSA101+ Immune checkpoint inhibitor | IMSA101 administered by intra-tumoral (IT) injection on Day 1 of Weeks 1, 2, and 3 for Cycle 1 and on Day 1 of Weeks 1 and 3 for all subsequent cycles. | Ⅰ/Ⅱ | NCT04020185 |  |
| BMS-986301 | Advanced solid tumors | BMS986301 + Nivolumab + Ipilimumab | IM; IT; IV; Specified dose on specified days. | Ⅰ | NCT03956680 |  |
| ONM-501 | Advanced Solid Tumors and Lymphomas | ONM-501 + Cemiplimab | IT; Administer intratumoral injection once a week for a total of 3 consecutive weeks. | Ⅰ | NCT06022029 |  |
| SNX281 | Advanced Solid Tumors and Lymphomas | SNX281 + Pembrolizumab | IV; Days 1, 8, and 15 in Cycle 1 and on Day 1 of each subsequent cycle thereafter of each 21-day cycle for up to 6 cycles | Ⅰ | NCT04609579 |  |
| ONO-7914 | Advanced/metastatic solid tumors | ONO-7914 + Nivolumab | Specified dose on specified days | Ⅰ | NCT06535009 |  |
| GSK3745417 | Refractory solid tumors | GSK3745417 + Dostarlimab | Follow an dose-escalation cohort escalation scheme | Ⅰ | NCT03843359 |  |
| SR-8541A | Advanced/metastatic solid tumors | SR-8541A + nivolumab or pembrolizumab | PO; Follow an accelerated titration dose (ATD) escalation scheme | Ⅰ | NCT06063681 |  |
| TXN10128 | Advanced/metastatic solid tumors | TXN10128 + Irinotecan or Paclitaxel | PO; This study includes a dose-escalation part and a dose-expansion part | Ⅰ | NCT05978492 |  |
| RBS2418 | Advanced/metastatic solid tumors | RBS2418+ Pembrolizumab | PO; Treatment Group A-1: 3 subjects per cohort, starting at 100 mg BID, with 100% dose increments (dose doubling) per subsequent cohort up to 800 mg BID. | Ⅰa/b | NCT05270213 |  |

Note: IT, Intratumoral injection; IV, Intravenous injection, IM, Intramuscular injection; PO: Peros.

**Supplementary Table 2 Clinical drugs for cGAS-STING pathway**

| **Compound** | **Indication** | **Intervention/Treatment** | **Phase** | | **NCT** |
| --- | --- | --- | --- | --- | --- |
| CDK-002 | Advanced/metastatic solid tumors | IT; Follow an dose-escalation cohort escalation scheme | Ⅰ/Ⅱ | NCT04592484 | |
| IMSA101 | Advanced/metastatic solid tumors | IT | I/IIa | CTR20211689 | |
| HG381 | Advanced solid tumors | IV; Subjects will receive HG381 IV at every one week intervals (Q1W) | Ⅰ | NCT04998422/ CTR20211765 | |
| KL340399 | Advanced solid tumors | IT; KL340399 weekly on Days 1, 8 and 15 on repeated 21-day cycles in escalating doses. | Ⅰ | NCT05549804 | |
| CRD3874-SI | Relapsed/​Refractory AML | IV infusion over one hour Strength: 6 mg/mL Schedule: Cycle: once weekly infusion x 4 (Days 1, 8, 15 and 22) over 28-day for Cycles 1 and 2. For Cycle 3 and onward, weekly infusion x 3 (Days 1, 8 and 15) | Ⅰ | NCT06626633 | |
|  | Advanced/metastatic solid tumors | IV; Cycle 1 & 2: once weekly infusion x4 (Days 1, 8, 15, 22) over 28-day cycle. Cycle 3 onwards: weekly infusion x 3 (Days 1, 8, 15) over 28-day cycle | Ⅰ | NCT06021626 | |

Note: IT, Intratumoral injection; IV, Intravenous injection, IM, Intramuscular injection; PO: Peros.
